# West Nile Virus Neurologic Disease in Humans, South Africa, September 2008–May 2009

**DOI:** 10.3201/eid1812.111208

**Published:** 2012-12

**Authors:** Dewald Zaayman, Marietjie Venter

**Affiliations:** Author affiliations: University of Pretoria, Pretoria, South Africa (D. Zaayman, M. Venter);; National Institute for Communicable Diseases, Johannesburg, South Africa (M. Venter)

**Keywords:** West Nile virus, WNV, neurologic disease, humans, South Africa, viruses, mosquito-borne, Flaviviridae, fever, viruses

## Abstract

We investigated West Nile virus (WNV) as a possible disease etiology in persons hospitalized in South Africa. Of 206 specimens tested, 36 had WNV neutralizing antibodies, significantly more than in similar earlier serosurveys. Seven probable acute WNV cases were identified, suggesting WNV may be overlooked as an etiology of severe disease in South Africa.

West Nile virus (WNV), a mosquito-borne flavivirus (1), is a reemerging pathogen of global concern (2). Febrile illness occurs in ≈20% of WNV-infected persons; neurologic complications (e.g., meningitis, encephalitis, flaccid paralysis) occur in <1% (3).

Detection of IgM in serum or cerebrospinal fluid (CSF) is the preferred method for diagnosing WNV infection; however, because of cross-reactivity between flaviviruses, positive results should be confirmed by virus neutralization assay. Early WNV infection can be diagnosed by PCR and virus isolation (4), but success has been limited in diagnosing more advanced disease with these techniques.

WNV is endemic to southern Africa. In 1947, one of the largest WNV epidemics recorded occurred in the Karoo region of South Africa (5), and another occurred in combination with a Sindbis virus epidemic in 1983–84 in the Witwatersrand–Pretoria region of South Africa. The most recent seroprevalence data for WNV in the Pretoria region of South Africa is from the 1970s (reviewed in [6]). To update that information, we determined whether WNV is being overlooked as a possible cause of disease in persons hospitalized in South Africa. The University of Pretoria Research Ethics Committee approved this study.

## The Study

Serum and CSF samples were obtained from the National Health Laboratory Service, Thswane Academic Division, Thswane, South Africa, which serves public sector hospitals in northern South Africa. To select samples for testing, we reviewed laboratory submission requests for patients with clinical conditions consistent with WNV infection: fever, headache, rash, or neurologic signs (7,8). A total of 206 patient samples (15 CSF and 191 serum) were selected. During September 2008–May 2009, we screened samples for the presence of WNV by using real-time, reverse-transcription PCR (rRT-PCR) (9), virus neutralization assay, and IgM ELISA.

We detected WNV neutralizing antibodies in serum and CSF samples by using a modified method (10). In brief, we mixed 50% tissue culture infective doses of Kunjin virus strain MRM61C (100 U/mL) in 2% fetal bovine serum (Invitrogen, Carlsbad, CA, USA) with 2-fold dilutions of heat-inactivated patient serum (1:10–1:640) in equal volumes and incubated the mixture for 1 h at 37°C in 5% CO_2_. We then added 1 volume of Vero cells (1 × 10^5^ cells/mL) in 2% fetal bovine serum, 100 U/mL of penicillin, and 100 μg/mL of streptomycin (Lonza, Basel, Switzerland) and incubated the mixture for 72 h at 37°C in 5% CO_2_. Samples were considered positive for WNV neutralizing antibodies if <25% of the cells/well displayed cytopathic effect. Comparative testing with Wesselsbron virus, a closely related flavivirus, did not show cross-reactivity within the parameters of what we considered positive.

Of the 206 specimens, 40 (19.42%, 95% CI 14.02%–24.82%) were positive for neutralizing antibodies. Of these, 36/191 serum samples had antibody titers of <160. The positive CSF samples (4/15) had antibody titers of 4 (Table 1). Positive serum samples were also tested by WNV IgM capture ELISA (Panbio; Alere, Sinnamon Park, QLD, Australia); 2 had positive results (Table 1).

**Table 1:** Characteristics and clinical information for 7 acutely ill patients with WNV infection, South Africa*

Sample no.	Date sample collected	Patient age, y/sex	Sample type	WNV antigen/antibody test			Clinical information
				AN (titer)	IgM	PCR	
4562	2008 Nov	45/M	CSF	Pos (4)	–	–	Suspected HIV encephalopathy or PML, TPHA neg, HIV neg, paraparesis
6208	2008 Nov	35/F	CSF	Pos (4)	–	–	Hepatomegaly, lymphadenopathy, fever, vomiting, epigastric pain, EBV IgM neg, EBV IgG pos, malaria neg, hepatitis neg
8785	2009 Jan	36/M	CSF	Pos (4)	–	–	Acute paresis of lower limbs, delirium, HSV-1 and HSV-2 neg (PCR), HTLV-1 neg, TPHA neg
3111	2009 Feb	5/M	CSF	Pos (4)	–	–	Meningitis, enterovirus pos (PCR)
0269	2009 Apr	11/M	Serum	Pos (40)	Pos	Neg	Rash, fever, *Brucella* neg (PCR), coxsackieviruses B1–B6 neg, CMV IgM neg, CMV IgG pos
0312	2009 Apr	26/M	Serum	Pos (80)	Pos	Neg	Severe headache, fever, suspected enterovirus, coxsackieviruses B1–B5 neg, *Rickettsia conorii* neg, EBV IgM neg
SAH 5238	2008 Oct	2/M	CSF	Neg	Neg	Pos	Decreased level of consciousness, rash, fever, meningitis, measles neg, mumps neg

*WNV, West Nile virus; AN, antibody neutralization; CSF, cerebrospinal fluid; pos, positive; –, insufficient sample for testing; PML, progressive multifocal leukoencephalopathy; TPHA, *Treponema pallidum* hemagglutination assay; neg, negative; EBV, Epstein-Barr virus; HSV-1 and -2, herpes simplex virus types 1 and 2; HTLV, human T-lymphotropic virus; CMV, cytomegalovirus.

Of the 206 specimens, 190 were of sufficient quantity to be subjected to RNA extraction (QIAamp Viral RNA Mini Kit; QIAGEN, Valencia, CA, USA) and subsequent WNV nested rRT-PCR (9). The presence of lineage 2 WNV RNA was identified in 1 CSF specimen and confirmed by sequencing (GenBank accession no. JX974605) (Figure).

**Figure:**
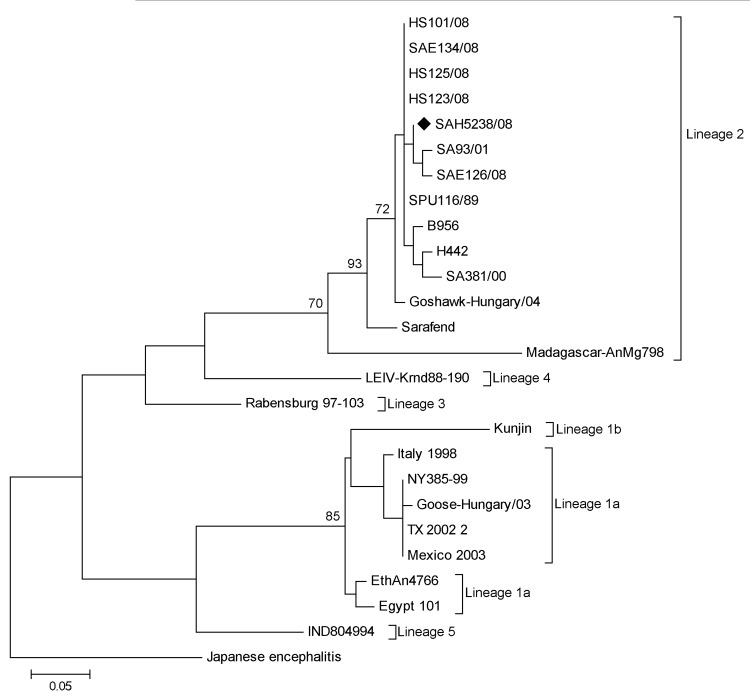
Maximum-likelihood tree of an ≈200-bp fragment of the nonstructural 5 gene of a reverse transcription PCR–positive West Nile virus (WNV) specimen SAH5238/08 (GenBank accession no. JX974605; black diamond) isolated from a human in South Africa in 2008. The tree shows the relationship of the strain to representative sequences of 5 WNV lineages, including 5 WNV lineage 2 strains isolated from horses in South Africa in 2008 (15). The scale bar indicates nucleotide substitutions per site. Bootstrap statistics of >70% are indicated on the tree branches. WNV strains (accession numbers): B956 (AY532665), SA381/00 (EF429199), SA93/01 (EF429198), SPU116/89 (EF429197), Goshawk-Hungary/04 (DQ116961), H442 (EF429200), Sarafend (AY688948), Madagascar AnMg798 (DQ176636), HS123/08 (FJ464376), HS101/08 (FJ464378), SAE126/08 (FJ464379), SAE134/08 (FJ464380), HS125/08 (FJ464377), Rabensburg97103 (AY765264), LEIV-Krnd88-190 (AY277251), Kunjin (D00246), Egypt101 (AF260968), EthAn4766 (AY603654), Italy1998 (AF404757), Goose-Hungary/03 (DQ118127), NY385-99 (EF571854), TX2002 (DQ164205), Mexico2003 (AY660002), IND804994 (DQ256376), Japanese encephalitis ( HM228921).

To evaluate the sensitivity of these molecular and serologic tests for diagnosing WNV in humans, we used the same methods to test 9 archived sequential serum samples in parallel (Table 2). The samples were from a patient with WNV encephalitis who became infected with neuroinvasive lineage 2 WNV strain in 2003 after a needlestick injury (11). Samples were collected 0–30 days after exposure. Initial symptoms developed on postexposure day 7 and persisted for 19 days; the patient completely recovered by day 26 (11). 

**Table 2:** Results of a time-trial experiment with serum samples from a WNV-infected person, South Africa*

Days after exposure to WNV	IgM ELISA†	Neutralization assay (titer)	Nested PCR
0	Neg	Neg	Neg
8	Neg	Neg	Pos
9	Neg	Neg	Pos
10	Neg	Neg	Neg
11	Neg	Neg	Neg
13	Neg	Pos (20)	Neg
16	Pos	Pos (40)	Neg
26	Pos	Pos (40)	Neg
30	Pos	Pos (80)	Neg

## Conclusions

We conducted a retrospective investigation of patients hospitalized with febrile illness or neurologic disease of unknown etiology in the Pretoria region of South Africa to determine whether some of the cases could be ascribed to WNV infection. Evidence of acute WNV infection was identified in samples for 7 patients (Table 1). For 2 of the patients, WNV infection was identified by the presence of IgM and neutralizing antibodies in serum samples; these patients had been hospitalized for febrile illness. For the other 5 patients, infection was identified by a WNV–positive (by PCR) CSF sample (1 patient) and by the presence of neutralizing antibodies in CSF samples (4 patients); these patients had been hospitalized for neurologic signs and symptoms.

The 4 patients with neutralizing antibodies in CSF all had severe neurologic complications (Table 1). Samples from these patients were insufficient for performing IgM testing; thus, WNV infection cannot be definitively determined. However, the presence of WNV neutralizing antibody in CSF samples plus acute clinical signs and symptoms of WNV infection provide a high index of suspicion for WNV infection in these patients. Factors such as increased blood–brain barrier permeability and the persistence of WNV antibodies long after infection may also serve as explanations for the presence of neutralizing antibodies in their CSF.

Nested rRT-PCR results and phylogenetic analysis confirmed the presence of lineage 2 WNV in the CSF sample from 1 patient (Figure); sequencing showed that the virus is closely related to 2 neuroinvasive WNV lineage 2 strains identified in South Africa (11,12) (Table 1). The low rate of PCR-positive cases was not entirely unexpected and may be explained by 2 factors: 1) PCR has limited success for detecting arboviruses because CSF contains low levels of virus and arbovirus-associated viremia is brief (13), and 2) false-negative test results may occur if samples are not properly stored to protect the integrity of potential viral RNA.

To evaluate the sensitivity of the 3 diagnostic methods used in our study, we conducted a time-trial experiment by using retrospective serum samples from a patient in whom WNV meningoencephalitis developed after a needlestick injury (11). Early samples (postexposure days 8 and 9) were positive for WNV by rRT-PCR only. Results for samples obtained ≥13 and ≥16 days after exposure were positive by neutralization assays and IgM ELISA, respectively (Table 2). Experiments with horses have indicated that WNV neutralizing antibody assays show a positive result earlier than IgM ELISAs; the reasons for this are undetermined (14). Although our time-trial experiment reflects findings from only 1 patient and should ideally be performed on a cohort, the results, considered with those from the studies in horses (14), may imply that some cases of WNV infection in humans and animals may be missed if IgM ELISA is the only serologic test used.

Using virus neutralization assays, we identified the presence of WNV antibodies in 36/204 serum samples from patients with febrile and neurologic illness in South Africa. This finding indicates that the patients were exposed to WNV. In addition, results were negative for the patients in our study who were tested for herpes simplex virus types 1 and 2, measles, mumps, and enteroviruses, and no other etiologic agent was found. Thus, infection with WNV should be included in the differential diagnosis of patients in this region with neurologic disease, especially considering the frequent detection of severe neurologic disease in horses in the region (15).

Our findings confirm that WNV is being overlooked as a cause of severe neurologic disease in South Africa, and they suggest a need for increased clinical awareness, enhanced prospective surveillance, and a more current serosurvey of WNV infection in humans. PCR may be a useful diagnostic method during early infection, but after seroconversion has taken place, serologic tests (e.g., IgM ELISA in conjunction with virus neutralization) are more likely to yield accurate results.
